# A Comparison Between Potassium Hydroxide (KOH) Microscopy and Culture for the Detection of Post-COVID-19 Rhino-Orbital-Cerebral Mucormycosis

**DOI:** 10.7759/cureus.47707

**Published:** 2023-10-26

**Authors:** Saqib Hasan, Prashant Gupta, Diksha Shukla, Gopa Banerjee

**Affiliations:** 1 Microbiology, King George's Medical College, Lucknow, IND

**Keywords:** mucorales, diagnostic accuracy, fungal culture, koh microscopy, post covid rocm

## Abstract

Background and objective

Mucormycosis is an emerging and serious angioinvasive infection caused by filamentous fungi related to the order of Mucorales and the class of Mucormycetes. There was a marked increase in the number of cases of mucormycosis in India following the second wave of the coronavirus disease 2019 (COVID-19) pandemic in the year 2021. In this study, we aimed to compare potassium hydroxide (KOH) microscopy with culture for the detection of post-COVID-19 rhino-orbital-cerebral mucormycosis (ROCM).

Materials and methods

The KOH microscopy was performed with a 10% or 20% KOH-mounted slide with specimens collected from suspected cases of post-COVID-19 ROCM. Simultaneously, the culture was done on Sabouraud dextrose agar (SDA). These were incubated at 37 ℃ and 25 ℃ for 28 days. Diagnostic parameters were calculated by comparing KOH with gold standard culture.

Results

KOH mount was positive for broad aseptate fungal hyphae in 322 (54.1%) cases, while it was negative in 244 (41.0%) cases. KOH mount was positive for other fungi in 29 (4.8%) samples. The diagnostic accuracy of the KOH mount for Mucorales was 70.3%. KOH mount had a sensitivity of 84.9%, specificity of 61.5%, positive predictive value (PPV) of 56.9%, and negative predictive value (NPV) of 87.2%.

Conclusions

Based on our findings, the KOH microscopy positivity rate was higher in tissue samples compared to nasal swabs, with a sensitivity of 84.9%, specificity of 61.5%, PPV of 56.9%, and NPV of 87.2%. The overall diagnostic accuracy of the KOH mount for Mucorales was 70.3%.

## Introduction

Mucormycosis is a life-threatening fungal infection, which was reported in significantly high numbers during the coronavirus disease 2019 (COVID-19) pandemic in India [1]. It is also classified as an opportunistic fungal illness with a high incidence in India (incidence rate of 140 per million) and a high case fatality rate. A high risk of mortality (up to 50%) has been found in patients infected with the Mucorales species of fungus [2].

Rhino-orbital-cerebral mucormycosis (ROCM) is a rare, acute, and severe fungal infection that occurs in several immunocompromised states, including diabetes, which happens to be the most prevalent (60-81%) risk factor [3]. Following the inhalation of fungal spores, the disease process is initiated in the nasal/sinus mucosae and then rapidly spreads to adjacent tissues, including the orbit and occasionally the brain. ROCM is associated with extremely high residual morbidity and mortality due to the fungus's angioinvasive nature, resulting in vascular blockage and severe tissue necrosis [4].

The diagnosis of Mucorales is very challenging but also essential for the management of these patients [5]. Traditional diagnoses are based on histopathologic and cultural analyses of the afflicted tissue. However, getting a precise result takes time and requires a lot of work. An immediate and accurate diagnosis is often warranted in these cases since the symptoms are vague, and the disease rapidly progresses with frequent fatal outcomes [6]. Potassium hydroxide (KOH) microscopy can provide a rapid provisional report.

After the second wave of COVID-19, there was a surge in the cases of mucormycosis in India. We collected various samples for evaluation, which enabled us to analyze varied samples for broad aseptate fungal hyphae. In light of this, we planned this study to assess the KOH microscopy mount's sensitivity, specificity, and diagnostic accuracy.

## Materials and methods

Study setting and ethical consideration

This cross-sectional study was carried out in the Department of Microbiology, King George's Medical University, Lucknow over a period of 12 months from May 2021 to April 2022. The study was approved by the Ethics committee of King George's Medical University (ref. code: PGTSC-IIA/P29). A total of 595 cases were enrolled from patients admitted to the hospital by applying the inclusion criteria after obtaining written informed consent (in patients' local language).

Study design

Clinically suspected patients were selected based on the inclusion criteria. Specimens selected were subjected to microscopy and a KOH mount was prepared. Simultaneously, samples were inoculated on Sabouraud dextrose agar (SDA). Cultures were kept for a maximum of 28 days if they were not positive for Mucorales. Sensitivity, specificity, positive predictive value (PPV), and negative predictive value (NPV) were calculated by comparing KOH with gold standard culture.

Inclusion criteria

COVID-19 and post-COVID patients presenting with any of the following clinical and radiological findings were included in the study: (1) nasal symptoms like stuffiness, nasal discharge - foul smell, epistaxis, (2) facial pain, facial edema, facial paraesthesia or anesthesia, (3) dental pain, (4) ophthalmic symptoms like pain around the eye, proptosis, diplopia, diminution or loss of vision, and (5) supportive diagnostic nasal endoscopy and/or GAD-MRI/CT scan findings like mucosal thickening of sinuses, cerebral edema, cerebral inflammation, and cerebral infarcts.

Sample collection

The following specimens were collected from the patient (one specimen from each patient), depending upon the clinical condition of the patient and the suspected site of infection: nasal swab, nasal tissue, maxillary tissue, vitreous fluid, conjunctival swab, and orbital contents.

Direct microscopic examination

A portion of the sample was taken on a grease-free slide for microscopy. The specimen was then subjected to KOH wet preparation of various concentrations (10%, 20%) depending upon the type of specimen for fungal element presence. The prepared slide was later examined under low (10x) and high (40x) objective lenses for the presence of fungal hyphae. The fungal hyphae appear as hyaline, broad aseptate branching at a 90-degree angle.

Culture

Simultaneously, irrespective of the presence of fungal hyphae on the KOH mount, the culture was performed in two sets of media: SDA with chloramphenicol 50 mg/L. These were incubated at room temperature at 37 ℃ and 25 ℃ for 28 days.

Statistical analysis

Data were entered into Microsoft Excel and analyzed using the statistical software IBM SPSS Statistics version 26 (IBM Corp., Armonk, NY).

## Results

KOH mount was positive for broad aseptate fungal hyphae in 322 (54.1%) cases, while it was negative in 244 (41.0%) cases. KOH mount was positive for septate hyphae or other fungi in 29 (4.8%) samples. Nasal tissue showed a high KOH mount positivity rate (83.3%) followed by maxillary tissue (73.3%), whereas nasal swabs showed the lowest KOH positivity rate (23.6%). On the other hand, the culture positivity rate was lower than KOH in the tissue sample (Table 1). Table 2 shows the KOH microscopy findings for fungal elements.

**Table 1:** Positivity of KOH microscopy and culture with respect to the type of sample (N=595) KOH: potassium hydroxide

Sample type	No. of cases	Percentage	KOH positive for Mucorales, n (%)	Culture positive for Mucorales, n (%)	KOH:culture ratio
Nasal swab	235	39.4	38 (16.1)	56 (23.8)	0.67
Nasal crust	31	5.2	25 (80.6)	21 (67.7)	1.19
Nasal tissue	228	38.3	190 (83.3)	112 (49.1)	1.69
Maxillary tissue	90	15.1	66 (73.3)	12 (13.3)	5.5
Exenterated orbital content	5	0.8	1 (20)	1 (20)	1
Vitreous fluid/tap	5	0.8	2 (40)	2 (40)	1
Conjunctival swab	1	0.2	0 (0)	0 (0)	-

**Table 2:** KOH microscopy findings for fungal elements (N=595) KOH: potassium hydroxide

S. no.	KOH microscopy	No. of specimens	Percentage
1	KOH negative	244	41.0
2	KOH positive for Mucorales	322	54.1

As shown in Table 3, a total of 532 samples were considered, with KOH findings associated with contaminated cultures and culture findings where KOH indicated the presence of other fungi excluded.

**Table 3:** KOH performance characteristics in comparison to culture for COVID-19-associated ROCM (n=532) k: 0.422 (moderate agreement) KOH: potassium hydroxide; COVID-19: coronavirus disease 2019; ROCM: rhino-orbital-cerebral mucormycosis

	Culture positive	Culture negative	Total
KOH positive	169	128	297
KOH negative	30	205	235
Total	199	333	532

The diagnostic accuracy of the KOH mount for Mucorales was 70.3%. KOH mount had a sensitivity of 84.9%, specificity of 61.5%, PPV of 56.9%, and NPV of 87.2% (Table 4).

**Table 4:** Diagnostic parameters of KOH mount in comparison to culture KOH: potassium hydroxide; PPV: positive predictive value; NPV: negative predictive value

Sensitivity	Specificity	PPV	NPV	Diagnostic accuracy
84.92	61.56	56.90	87.23	70.3

## Discussion

In our study, the highest number of positive cases by KOH microscopy was found in tissue samples, which was significantly higher when compared to nasal swabs and crust. These findings are similar to those observed in previous studies [7]. Based on these findings, we recommend that tissue samples be always preferred over swabs for the microscopy of a suspected case of ROCM.

Most of the Mucorales grow rapidly (three to seven days) on ordinary fungal culture media such as Sabouraud agar and potato dextrose agar incubated at 25 ℃ to 30 ℃. However, the growth in cultures from the samples was not 100%. Walsh et al. and Mohanty et al. observed positivity of below 50% in cultures [8,9]. In our study, the positivity rate was only 34.2%. Culture positivity also depended on the type of sample. The culture had the highest positivity rate in nasal crusts (67.7%) followed by nasal tissues (49.1%) and nasal swabs (23.8%) (Table 1). However, the KOH vs. culture ratio in the tissue samples was higher (1.69) when compared to the swab samples (0.67) (Table 1).

Low positivity in culture may be attributed to the friable nature of hyphae. They may also get damaged during tissue manipulation processes like homogenization and grinding. Culture positivity may also be affected by the portion of the tissue taken for culture. The portion of tissue with no fungal element may not result in culture positivity [9,10]. The reason for microscopy negativity and culture positivity may be due to the fact that the portion of tissue that was processed did not have fungal hyphae. Contamination was ruled out with the use of repeated samples, and these were compared with clinical and radiological findings.

Invasive fungal infection is a major cause of high mortality and morbidity, particularly in immunocompromised patients. Hence, rapid diagnosis is required for any intervention. KOH-based direct microscopy is a rapid and useful method to detect Mucorales infection, especially in tissue samples. In our study, KOH microscopy had a moderate agreement (k: 0.422) with culture; it had a high sensitivity (84.92%), specificity (61.56%), and very good NPV (87.23%) when compared with gold standard cultures. Similar findings have been reported by Mohanty et al. (2021). The low PPV observed (56.90%) could be due to the low growth of Mucorales on culture [8]. Table 5 summarizes the findings of various similar studies in the literature in comparison with the present study.

**Table 5:** Varying findings with regard to different fungi found in various studies in the literature when compared to the present study PPV: positive predictive value; NPV: negative predictive value

	Bhabor et al. (Mucorales) [11]	Begari et al. (onychomycosis) [12]	Dass et al. (onychomycosis) [13]	Levitt et al. (tinea pedis) [14]	Present study
Sample size	230	102	150	460	595
Sensitivity	67.31%	81.81%	83.02%	73.30	84.9%
Specificity	87.79%	92.86%	70.01%	42.5%	61.5%
PPV	76.92%	69.23%	60.27%	46.6%	56.9%
NPV	81.62%	83.33%	88.31%	69.9%	87.2%

The findings by Bhabor et al. contrast with the present study, and it may be due to the smaller sample size and the fact that only tissue/scraping was taken by them. In contrast, in the present study, all types of samples were taken.

Limitations of the study

We feel that incorporating histopathological examination and PCR analysis for fungi could have added significant value to the study findings.

## Conclusions

Based on our findings, the highest number of positive cases by KOH microscopy was found in tissue samples, which was significantly higher when compared to nasal swabs and crusts. Hence, tissue samples should be preferred for KOH microscopy, and well-trained technical staff are required for isolation from tissue culture. The diagnostic accuracy of the KOH mount for Mucorales was 70.3%. KOH mount had a sensitivity of 84.9%, specificity of 61.5%, PPV of 56.9%, and NPV of 87.2%.

## References

[1] Sharma R, Kumar P, Rauf A (2022). Mucormycosis in the COVID-19 environment: a multifaceted complication. Front Cell Infect Microbiol.

[2] Prakash H, Chakrabarti A (2021). Epidemiology of mucormycosis in India. Microorganisms.

[3] Yohai RA, Bullock JD, Aziz AA, Markert RJ (1994). Survival factors in rhino-orbital-cerebral mucormycosis. Surv Ophthalmol.

[4] Parfrey NA (1986). Improved diagnosis and prognosis of mucormycosis. A clinicopathologic study of 33 cases. Medicine (Baltimore).

[5] Skiada A, Lass-Floerl C, Klimko N, Ibrahim A, Roilides E, Petrikkos G (2018). Challenges in the diagnosis and treatment of mucormycosis. Med Mycol.

[6] Acosta-España JD, Voigt K (2022). Mini review: risk assessment, clinical manifestation, prediction, and prognosis of mucormycosis: implications for pathogen-and human-derived biomarkers. Front Microbiol.

[7] Agarwal SS, Anand P, Rao S, Galhotra V (2022). Site-based comparative analysis of sample collection through direct biopsy and nasal swabs for early diagnosis of post-COVID rhinomaxillary fungal infection using potassium hydroxide mounting: a retrospective cohort study. J Maxillofac Oral Surg.

[8] Mohanty A, Gupta P, Arathi K (2021). Evaluation of direct examination, culture, and histopathology in the diagnosis of mucormycosis: reiterating the role of KOH mount for early diagnosis. Cureus.

[9] Walsh TJ, Gamaletsou MN, McGinnis MR, Hayden RT, Kontoyiannis DP (2012). Early clinical and laboratory diagnosis of invasive pulmonary, extrapulmonary, and disseminated mucormycosis (zygomycosis). Clin Infect Dis.

[10] Lackner M, Caramalho R, Lass-Flörl C (2014). Laboratory diagnosis of mucormycosis: current status and future perspectives. Future Microbiol.

[11] Bhabhor U, Mistry Y, Mullan S (2022). Evaluation of sensitivity and specificity of direct microscopical examination of suspected mucor mycosis samples by potassium hydroxide (KOH) during Covid-19 pandemic era. Adv Infect Dis.

[12] Begari V, Pathakumari P, Takalkar AA (2019). Comparative evaluation of KOH mount, fungal culture and PAS staining in onychomycosis. Int J Res Dermatol.

[13] Dass SM, Vinayaraj E, Pavavni K, Pallam A, Rao MS (2015). Comparison of KOH, calcofluor white and fungal culture for diagnosing fungal onychomycosis in an urban teaching hospital, Hyderabad. Indian J Microbiol Res.

[14] Levitt JO, Levitt BH, Akhavan A, Yanofsky H (2010). The sensitivity and specificity of potassium hydroxide smear and fungal culture relative to clinical assessment in the evaluation of tinea pedis: a pooled analysis. Dermatol Res Pract.

